# Sequential drug release from dual-responsive scaffold with ultrasound-enhanced efficacy for infectious oral ulcer therapy

**DOI:** 10.1093/rb/rbag063

**Published:** 2026-03-26

**Authors:** Siyi Li, Xiang Zhang, Shunan Wang, Ruonan Jiang, WenTing Du, Yin Yi, Yu Shu, Ziliang Xiu, Panpan Liang, Pengfei Zhou, Xiaohong Wu

**Affiliations:** Stomatological Hospital of Chongqing Medical University, Chongqing 401147, China; Chongqing Key Laboratory of Oral Diseases and Biomedical Sciences, Chongqing Medical University, Chongqing 401147, China; Chongqing Municipal Key Laboratory of Oral Biomedical Engineering of Higher Education, Chongqing Medical University, Chongqing 401147, China; Stomatological Hospital of Chongqing Medical University, Chongqing 401147, China; Chongqing Key Laboratory of Oral Diseases and Biomedical Sciences, Chongqing Medical University, Chongqing 401147, China; Chongqing Municipal Key Laboratory of Oral Biomedical Engineering of Higher Education, Chongqing Medical University, Chongqing 401147, China; Stomatological Hospital of Chongqing Medical University, Chongqing 401147, China; Chongqing Key Laboratory of Oral Diseases and Biomedical Sciences, Chongqing Medical University, Chongqing 401147, China; Chongqing Municipal Key Laboratory of Oral Biomedical Engineering of Higher Education, Chongqing Medical University, Chongqing 401147, China; Stomatological Hospital of Chongqing Medical University, Chongqing 401147, China; Chongqing Key Laboratory of Oral Diseases and Biomedical Sciences, Chongqing Medical University, Chongqing 401147, China; Chongqing Municipal Key Laboratory of Oral Biomedical Engineering of Higher Education, Chongqing Medical University, Chongqing 401147, China; Stomatological Hospital of Chongqing Medical University, Chongqing 401147, China; Chongqing Key Laboratory of Oral Diseases and Biomedical Sciences, Chongqing Medical University, Chongqing 401147, China; Chongqing Municipal Key Laboratory of Oral Biomedical Engineering of Higher Education, Chongqing Medical University, Chongqing 401147, China; Stomatological Hospital of Chongqing Medical University, Chongqing 401147, China; Chongqing Key Laboratory of Oral Diseases and Biomedical Sciences, Chongqing Medical University, Chongqing 401147, China; Chongqing Municipal Key Laboratory of Oral Biomedical Engineering of Higher Education, Chongqing Medical University, Chongqing 401147, China; Stomatological Hospital of Chongqing Medical University, Chongqing 401147, China; Chongqing Key Laboratory of Oral Diseases and Biomedical Sciences, Chongqing Medical University, Chongqing 401147, China; Chongqing Municipal Key Laboratory of Oral Biomedical Engineering of Higher Education, Chongqing Medical University, Chongqing 401147, China; Stomatological Hospital of Chongqing Medical University, Chongqing 401147, China; Chongqing Key Laboratory of Oral Diseases and Biomedical Sciences, Chongqing Medical University, Chongqing 401147, China; Chongqing Municipal Key Laboratory of Oral Biomedical Engineering of Higher Education, Chongqing Medical University, Chongqing 401147, China; Stomatological Hospital of Chongqing Medical University, Chongqing 401147, China; Chongqing Key Laboratory of Oral Diseases and Biomedical Sciences, Chongqing Medical University, Chongqing 401147, China; Chongqing Municipal Key Laboratory of Oral Biomedical Engineering of Higher Education, Chongqing Medical University, Chongqing 401147, China; Stomatological Hospital of Chongqing Medical University, Chongqing 401147, China; Chongqing Key Laboratory of Oral Diseases and Biomedical Sciences, Chongqing Medical University, Chongqing 401147, China; Chongqing Municipal Key Laboratory of Oral Biomedical Engineering of Higher Education, Chongqing Medical University, Chongqing 401147, China; Stomatological Hospital of Chongqing Medical University, Chongqing 401147, China; Chongqing Key Laboratory of Oral Diseases and Biomedical Sciences, Chongqing Medical University, Chongqing 401147, China; Chongqing Municipal Key Laboratory of Oral Biomedical Engineering of Higher Education, Chongqing Medical University, Chongqing 401147, China

**Keywords:** infectious oral ulcers, sequential drug release, on-demand drug release, dual-responsive hydrogel scaffold

## Abstract

Oral aphthous ulcers are among the most prevalent mucosal disorders, with infectious oral ulcers severely compromising patients’ quality of life. This study introduces a dual-responsive (ultrasound- and pH-sensitive) hydrogel–microsphere scaffold for their precise, staged management. The scaffold, composed of a pH-sensitive chitosan hydrogel and ultrasound-responsive sodium alginate microspheres, demonstrated injectability, biocompatibility and sequential drug release. Specifically, ultrasound enhanced drug release and tissue penetration, improving therapeutic efficacy. The pH-responsive mechanism enabled targeted release of lidocaine (LID) and curcumin (CUR) at inflamed sites, which is expected to provide local anesthesia while promoting epithelial regeneration. Mechanistically, ultrasound stimulation activated PI3K-Akt and ECM-receptor interaction pathways, which were associated with enhanced cell migration, proliferation and drug responsiveness. These effects not only enhanced the therapeutic efficacy but also established a microenvironment favorable for tissue repair. Our platform integrates multimodal functionalities, including rapid release of the analgesic drug LID, followed by the sustained effects of CUR. Crucially, ultrasound facilitates this release process and enhances the therapeutic efficacy. This comprehensive approach modulates the local wound microenvironment, thereby creating favorable conditions for accelerating tissue repair. This work expands new avenues for stimulus-responsive drug delivery systems in the treatment of oral diseases and holds potential for clinical translation.

## Graphical Abstract

**Graphical Abstract:**
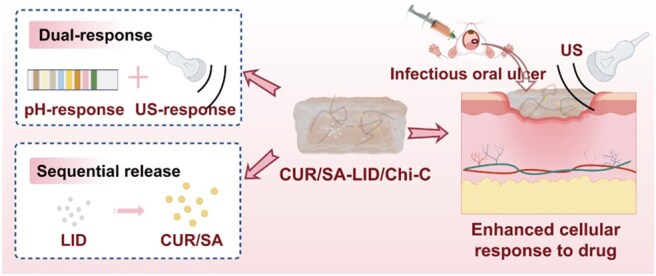

## Introduction

Oral aphthous ulcers are a common and frequently occurring mucosal disease, with a prevalence ranging from approximately 5% to 25% in the general population [1, 2]. Moreover, due to damage to the oral mucosal barrier, microorganisms can readily invade and cause infections in individuals [3] and interactions between microorganisms and keratinocytes can significantly influence the healing time of ulcers [4]. Critically, patients endure debilitating pain characterized by sharp, burning sensations exacerbated by mastication and speech—a primary symptom that frequently triggers nutritional compromise, sleep disruption and anxiety disorders. This profound pain burden severely diminishes quality of life, making effective analgesia not merely palliative but a therapeutic imperative [5]. Furthermore, secondary bacterial infections exacerbate both tissue damage and pain perception [6], creating complex clinical challenges in ulcer management.

Current management relies on topical antibiotics and glucocorticoids to control infection [7, 8], although antibiotic misuse has diminished treatment efficacy [9–11]. The misuse of antibiotics has driven increasing adoption of herbal medicines in oral ulcer treatment [12, 13]. Among them, Curcumin (CUR), a hydrophobic polyphenol extracted from the turmeric with demonstrated anti-inflammatory and antioxidant activities relevant to ulcer pathogenesis [14, 15]. However, CUR’s clinical utility remains constrained from its low solubility and the complex oral environment [16]. Therefore, improving CUR’s therapeutic efficacy is a critical challenge that needs to be addressed. Ultrasound-responsive technology not only addresses these limitations through noninvasive, spatiotemporally controlled energy delivery [17–19] but also enhances cell membrane permeability while promoting cellular proliferation and angiogenesis through mechanical stimulation [19–21]. When combined with antibacterial agents, ultrasound can also exert synergistic antimicrobial effects [22]. The therapeutic benefits of ultrasound arise from coordinated physical mechanisms, such as cavitation and mechanical effects, which improve tissue permeability and facilitate drug carrier penetration [18, 19]. These coordinated actions collectively improve drug delivery and therapeutic precision [23, 24], positioning ultrasound as a promising strategy for overcoming current treatment limitations in infectious oral ulcer management.

In infectious oral pathologies, dysregulated biofilm formation leads to microenvironmental acidification, perpetuating deleterious inflammatory responses [25]. Bacterial or viral infections elicit inflammatory responses that lower wound surface pH, thereby facilitating immune cell infiltration and pro-inflammatory cytokine release, while simultaneously inhibiting epithelial regeneration [26]. The critical role of pH in this pathology has made the wound microenvironment a key target for intervention. Emerging strategies, including gas-releasing nanoplatforms, antibacterial hydrogels and stem cell-based therapies, offer new options for managing chronic infected wounds and oral ulcers [27–29]. Among these, pH-controlled drug release is a well-established approach, proven to accelerate re-epithelialization, inhibit pathogenic colonization and regulate inflammatory processes [30, 31]. Consequently, pH-responsive hydrogel systems have emerged as a promising and targeted therapeutic strategy for infectious oral lesions by exploiting microenvironmental pH gradients to achieve controlled and precise drug release. This approach not only enhances therapeutic efficacy but also reduces both systemic and local adverse effects [32, 33].

In this study, a dual-responsive (ultrasound and pH) hydrogel-microsphere scaffold with sequential drug release capability was developed. We hypothesize that a drug delivery system capable of responding to both physicochemical (pH) and physical (ultrasound) stimuli can achieve precise, controllable and sequential release of therapeutic agents, thereby significantly improving treatment outcomes for infectious oral ulcers. This composite scaffold exhibits excellent injectability, biocompatibility and adhesion ability to the oral mucosa, enabling precise spatiotemporal regulation of drug delivery. To address the requirement for rapid pain relief in oral ulcer management [5], lidocaine (LID) was directly incorporated into the hydrogel matrix to achieve rapid and localized analgesia. In parallel, CUR was efficiently encapsulated in ultrasound-responsive sodium alginate (SA) microspheres using electrospray technology, and subsequently embedded into a highly adhesive, pH-responsive chitosan-catechol (Chi-C) hydrogel, ensuring sustained antibacterial, anti-inflammatory and tissue-regenerative effects. This intelligent delivery system was engineered to adapt dynamically to the complex oral environment. pH sensitivity enables selective drug release within inflamed tissues, simultaneously, ultrasound activation enhances drug efficacy, thereby potentiating therapeutic outcomes. This multimodal approach, already applied in chronic infected wound management [34], thus, provides a valuable new strategy for treating infectious oral ulcers. Collectively, this dual-stimuli-responsive platform not only improves the precision and efficacy of drug delivery but also highlights its significant potential for advancing personalized oral healthcare.

## Materials and methods

CUR (≥98% purity), Lidocaine hydrochloride monohydrate (LID, ≥98% purity), Chitosan (CS, Degree of deacetylation ≥95%, viscosity: 100–200 mPa·s, 1% in 1% acetic acid), Hydrocaffeic acid (HCA, 3,4-dihydroxy hydrocinnamic acid, ≥98% purity), 1-Ethyl-3-(3-dimethylaminopropyl)-carbodiimide hydrochloride (EDC, ≥98% purity), Calcium chloride anhydrous (CaCl_2_, AR, ≥96.0%) were purchased from Aladdin Co., Ltd. (Shanghai, China). SA (M/G ratio ∼ 1:2, Mw ∼ 30 kDa), Sodium periodate (NaIO_4_, AR, ≥99.5%), Ethoxylated hydrogenated castor oil (PEG-40, HLB: 13-14), N-Hydroxysuccinimide (NHS, ≥99% purity) were purchased from Macklin Biochemical Co., Ltd. (Shanghai, China). CCK-8 kit and live/dead staining kit were purchased from Beyotime Biotechnology Co., Ltd. (Shanghai, China). The apoptosis kit was bought from Elabscience Biotechnology Co., Ltd. (Wuhan, China). Luria–Bertani broth medium (LB) was purchased from Solarbio Science & Technology Co., Ltd. (Beijing, China), LB agar plates (without antibiotics) were purchased from Labgic Technology Co., Ltd. (Beijing, China). All other chemicals were of analytical grade.

### Cell lines and animals

Mouse mononuclear macrophages (RAW 264.7) were bought from Omega Technology Co., Ltd. (Jilin, China), NCTC clone 929 (L-929) was purchased from Noblebio Biotechnology Co., Ltd. (Zhejiang, China). Sprague–Dawley (SD) rats (6-week old) were obtained from Ensiweier Biotechnology Co., Ltd. (Chongqing, China) and were raised in a specific pathogen-free environment.

### Preparation of CUR/SA microspheres, Chi-C hydrogels and CUR/SA-LID/Chi-C

CUR solution was prepared first. 0.2 g of CUR was dispersed into 4.4 g of RH40 and stirred at 25°C under light protection for 3 h. A 2% w/v SA solution was prepared and the CUR solution was mixed with the SA solution at a volume ratio of 2:3. And then the prepared solution was loaded into a 5 mL injector with 25 G syringe needle and the solution was dripped into a 3% w/v CaCl_2_ solution by using electrospray technique. Microspheres were prepared using flow rate (0.120 mm/min), collection distance of 30 cm and applied voltage (20 kV). The microspheres were filtered, collected and placed in a lyophilizer for 48 h. The lyophilized microspheres were kept in a dark dry place and subsequently observed using a field-emission SEM (ZEISS GeminiSEM 300, Germany).

CS (504.2 mg, 3.0 mmol) was hydrated in ultrapure water with pH 5.0 adjusted with HCl to form a 1.0 wt% solution of CS. HCA (546.5 mg, 3.0 mmol) dissolved in 5.0 mL of ultrapure water was then added to the CS solution. EDC (575.1 mg, 3.0 mmol) and NHS (345.3 mg, 3.0 mmol) dissolved in a 50.0 mL mixture of ethanol and ultrapure water (1:1, v/ v) were then added dropwise to the CS/HCA solution. The reaction mixture was stirred vigorously at room temperature for 10.0 h, maintaining its pH value at 5.0. To remove unreacted residues, the prepared Chi-C solution was dialyzed in pH 3–4 ultrapure water for 2 days, followed by half a day of neutralization in ultrapure water. The resulting solution was freeze-dried to obtain Chi-C solid, which was stored in a refrigerator.

And then Chi-C solution (2 wt%) was configured, 2% w/v LID powder and different ratios of CUR/SA microspheres were added, crosslinking was done by adding 5% w/v solution of NaIO_4_, mixing and shaking until the formation of CUR/SA-LID/Chi-C hydrogel-microsphere composite drug-carrying scaffolds. The lyophilized Chi-C and CUR/SA-LID/Chi-C samples were observed under field-emission scanning electron microscope.

### Fourier transform infrared spectroscopy

Chi-C lyophilized hydrogels and their raw materials were structurally characterized by Fourier transform infrared (FTIR) technique. Each sample was pressed mixed with potassium bromide and fully milled to make a homogeneous powder and pressed. The samples were scanned and analyzed in the wave number range of 4000–500 cm^−1^ using a FTIR spectroscopy (Thermo Scientific Nicolet iS5, USA).

### Rheological testing

The rheological properties of the hydrogels were tested by a rotational rheometer (MCR 302, Anton Paar, China). The test temperature was 25°C, the fixture diameter was 20 mm and scans were performed in the range of 0–100 s^−1^ shear rate to record the viscosity-shear rate curve. As well as dynamic frequency scans from 0.1 rad/s to 100 rad/s were performed to record the storage modulus (*G*′) and loss modulus (*G*″).

### Viscosity test

The lap-shear test was performed on a universal testing machine (E43, MTS Instruments, USA). Fresh pig skin of size 20 mm × 60 mm was wetted with PBS at pH 5 and the mixed hydrogel that had not yet been crosslinked was coated on the surface of the pig skin, gently kneaded to cover uniformly, with a lap area of 20 mm × 25 mm and the lap-shear test was carried out after 1 h.

### *In vitro* drug release from hydrogels at different pH

Five-hundred microliters of hydrogels loaded with 3 mg LID (500 μL) were immersed in 1 mL PBS (pH 5, 6.5, 7.4) at 37°C with shaking (60 rpm). At 2, 4, 6, 8 and 10 h, 0.5 mL supernatant was collected for analysis at 261 nm and replaced with fresh PBS. Separately, CUR/SA microspheres containing 0.5 mg CUR were incorporated into hydrogels and placed in 1 mL PBS with 0.5% SDS (pH 5, 6.5, 7.4). Supernatant (0.5 mL) was collected at 6, 12, 24, 48, 72, 96, 120, 144 and 168 h and analyzed at 425 nm using a multimode plate reader (PerkinElmer).

### *In vitro* drug release from hydrogels at different ultrasound sound intensities

CUR/SA microspheres and hydrogel-microsphere scaffolds were prepared according to previous methods. The treatment was carried out using a fully digital ultrasound therapeutic apparatus (Welld, Model WB9-100, China; Frequency: 1.0 MHz; Pulse Repetition Interval: 10 ms; Exposure Duration: 5 min per treatment session; Transducer Contact Area: 5 cm^2^). For CUR/SA microspheres, ultrasound (0.25 or 0.55 W/cm^2^) was applied at 2 and 4 h, with cumulative release measured at 1, 2, 3, 4, 6, 10, 24, 48 and 72 h. In contrast, for CUR/SA-LID/Chi-C hydrogel-microsphere scaffolds, ultrasound (0.25 or 0.55 W/cm^2^) was applied every 24 h and release was measured at 6, 12, 24, 48, 72, 96, 120, 144, 168 and 192 h. Additionally, to assess the impact of pH and ultrasound, experiments were conducted in pH 5, 6.5 and 7.4 buffers with a fixed ultrasound intensity of 0.55 W/cm^2^. Ultrasound was applied every 24 h and measurements were taken at 6, 12, 24, 48 and 72 h.

### *In vitro* transdermal diffusion study

Prepared ex vivo pig skin was mounted on a Franz diffusion cell. PBS buffer (pH 6.5) containing 0.5% SDS was used as the receptor medium under constant temperature and stirring. A hydrogel containing 10 mg of CUR/SA microspheres was applied to the skin for two groups: CUR/SA-Chi-C+US (0.55 W/cm^2^, 5 min at *t* = 0 h, 24 h) and CUR/SA-Chi-C. Samples were collected from the receptor chamber at 6, 12, 24 and 48 h, replaced with fresh medium and then analyzed for drug concentration to calculate cumulative permeation and plot the curve.

### *In vitro* cytotoxicity and biocompatibility

The cytotoxicity of hydrogel-microsphere scaffolds containing LID and CUR was tested with the CCK-8 assay. L-929 cells and HaCaT cells were seeded in 96-well plates (1 × 10^4^ cells/cm^2^) and cultured with material extracts for 24 h. Groups included the untreated control, US, CUR/SA-LID/Chi-C and CUR/SA-LID/Chi-C+US. After 24 h, cell metabolic activity was assessed by measuring absorbance at 450 nm.

To further evaluate hydrogel biocompatibility, apoptosis was analyzed using an apoptosis detection kit. L-929 cells were co-cultured with each group for 24 h, then, stained to distinguish living and dead cells. Apoptosis was quantified using Annexin V-FITC/PI staining (V-FITC 1:100, PI 1:50), followed by flow cytometry analysis (BD Biosciences, USA).

### Scratch and transwell experiments

L929 cells were seeded in six-well plates. At 90% confluence, a scratch was made with a 200 µL pipette tip. After PBS washes, experimental groups received serum-free medium with material extract or ultrasound-treated products; controls received serum-free medium only. Scratch areas were imaged at 0 and 24 h (Nikon Eclipse Ts2) and wound closure was quantified using ImageJ.

For Transwell assays (8 µm pores), L929 cells (2 × 10^5^ cells/mL, 100 µL) were seeded in the upper chamber; the lower chamber contained 200 µL complete medium with 10% FBS. After 24 h incubation, nonmigrated cells were removed. Migrated cells were fixed with 4% paraformaldehyde (30 min), stained with 0.1% crystal violet (20 min), imaged (Nikon Eclipse Ts2) and counted with ImageJ. Migration rates were calculated relative to control.

### Antioxidant assay

PITO free radical scavenging capacity was determined. Each experimental group was placed in 1 mL of PITO solution and incubated at 37°C for 2 h. The absorbance of the resulting solution was measured at a wavelength of 557 nm and the inhibition rate was determined. The percent antioxidant activity was calculated according to the following formula: antioxidant activity (%) = [(ABSDPPH − ABSsample)/ABSDPPH] × 100%.

### ROS scavenging assay

The DCFH-DA fluorescent probe was used to assess ROS scavenging in different treatment groups. L929 cells were seeded in 96-well plates. After 24 h of treatment, 1 mM H_2_O_2_ was added for 30 min to induce ROS production. Supernatants were, then, discarded, and serum-free DMEM containing DCFH-DA was added and incubated for 20 min in the dark. Cells treated with H_2_O_2_ alone served as positive control; untreated cells served as negative control. After three PBS washes, intracellular fluorescence intensity was immediately observed and recorded using a fluorescence microscope (Nikon Eclipse Ts2).

### *In vitro* anti-inflammatory activity assay

RAW 264.7 cells (5 × 10³/well) were seeded in 96-well plates for 24 h. Inflammation was induced with 10 μg/mL LPS for 2 h, followed by application of hydrogel extracts. After 24 h, cell viability was assessed using CCK-8 assay.

To further investigate anti-inflammatory effects, total RNA was extracted from LPS-activated RAW 264.7 cells (control and CUR/SA-LID/Chi-C+US groups) using TRIzol. RNA purity and concentration were measured with a NanoDrop spectrophotometer (Thermo Scientific, USA). cDNA was synthesized from 2 μL RNA using a PrimeScript™ RT kit. q-PCR was conducted on a CFX96 system (Bio-Rad Laboratories, Inc., Hercules, CA, USA) using TB Green^®^ Premix. Expression of CD206, TNF-α, IL-10, AKT and PI3K was measured, with GAPDH as the reference gene. Relative expression was calculated by the 2^–ΔΔCt method, and all experiments were performed in triplicate.

### Antimicrobial assay

*S. aureus* and *E. coli* were used as representative strains for antimicrobial activity testing. Activated strains were inoculated in LB liquid medium and incubated at 37°C with shaking for 6 h to logarithmic phase. Bacterial suspension (1 mL, ∼1 × 10^8^ CFU/mL) was co-cultured with different groups of hydrogel-microsphere scaffolds for 6–8 h at 37°C. Then, 100 μL of the mixture were serially diluted and spread on LB agar plates to count colony-forming units (CFU) after 12 h incubation. Additional co-cultured scaffolds were subjected to crystal violet staining for biofilm quantification and live/dead bacterial staining for viability assessment.

### RNA sequencing (RNA-Seq) and data analysis

To clarify the effects of the materials at the transcriptomic level, RNA sequencing was performed. RAW 264.7 cells were seeded (6 × 10^5^ cells/mL) in 6-well plates and cultured for 24 h. Following 10 μg/mL LPS stimulation to induce pro-inflammatory polarization, the medium was replaced with DMEM containing CUR/SA-LID/Chi-C, with or without ultrasound treatment, and co-cultured for another 24 h. Total RNA was extracted using TRIzol and subjected to quality control. Qualified samples were sequenced by Wuhan Igenebook Biotechnology Co., Ltd. Differential expression, GO and KEGG enrichment analyses were subsequently conducted.

### Establishment and treatment of oral ulcer model in rats

The animal experimental protocols involved in this study were reviewed and approved by the Research Ethics Committee of The Affiliated Hospital of Stomatology, Chongqing Medical University [Approval No.: REC #COHS-REC-2024 (LSNo. 146)]. SD rats (6-week old) were selected and acclimatized in a standard environment for 7 days, followed by contacting the buccal and lingual ventral mucosa with 50% (v/v) glacial acetic acid-impregnated circular pieces of filter paper (6 mm in diameter) for 60 s to induce ulcer formation, and 10 μL of *S. aureus* suspension was added to the wound surface (1 × 10^8^ CFU/mL) to establish the infection model. At 24 h post-modeling, rats were randomly divided into three groups (*n* = 5/group): untreated control, CUR/SA-LID/Chi-C and CUR/SA-LID/Chi-C+US. Wound characteristics were observed daily, and ulcer area and healing rate were quantitatively assessed from Day 0 to 5.

### *In vivo* bacterial growth assay

To assess the post-treatment bacterial viability and growth potential, the buccal and lingual ventral mucosa were wiped three times using a sterilized swab and immediately placed in a sterile centrifuge tube containing 5 mL of LB liquid medium 24 h after the intervention. The sample tubes were processed in an ultrasonic cleaner for 5 min, after which the swabs were discarded and the bacterial suspensions were incubated at 37°C in a constant temperature incubator for 12 h. The bacterial growth was quantified by measuring the absorbance value of the culture solution at 600 nm using a multimode plate reader.

### Histological examination

After euthanizing the rats, ulcer tissue samples were harvested, fixed, dehydrated and embedded. Hematoxylin and eosin (H&E) staining assessed epithelial architecture, while Masson trichrome staining evaluated collagen deposition. H&E staining was also performed on heart, kidney, spleen, lungs and liver four weeks postoperatively to detect pathological changes. Immunofluorescence staining assessed CD31, α-SMA and Ki67 expression. Images were captured using an inverted fluorescence microscope (EclipseTs2, Nikon, Japan) and quantitatively analyzed with ImageJ software.

### Statistical methods

Statistical analyses were performed using GraphPad Prism version 8. Quantitative results are presented as the mean ± SD. Inter-group comparisons were assessed via Student’s *t*-tests, while multiple group analyses were carried out with one-way ANOVA followed by Tukey’s *post hoc* test. A *P* value of less than 0.05 was regarded as statistically significant, with increasing asterisks denoting higher levels of significance (*P < 0.05, **P < 0.01, ***P < 0.001, ****P < 0.0001).

## Result and discussion

### Synthesis and characterization of Chi-C hydrogels

**Figure 1:**
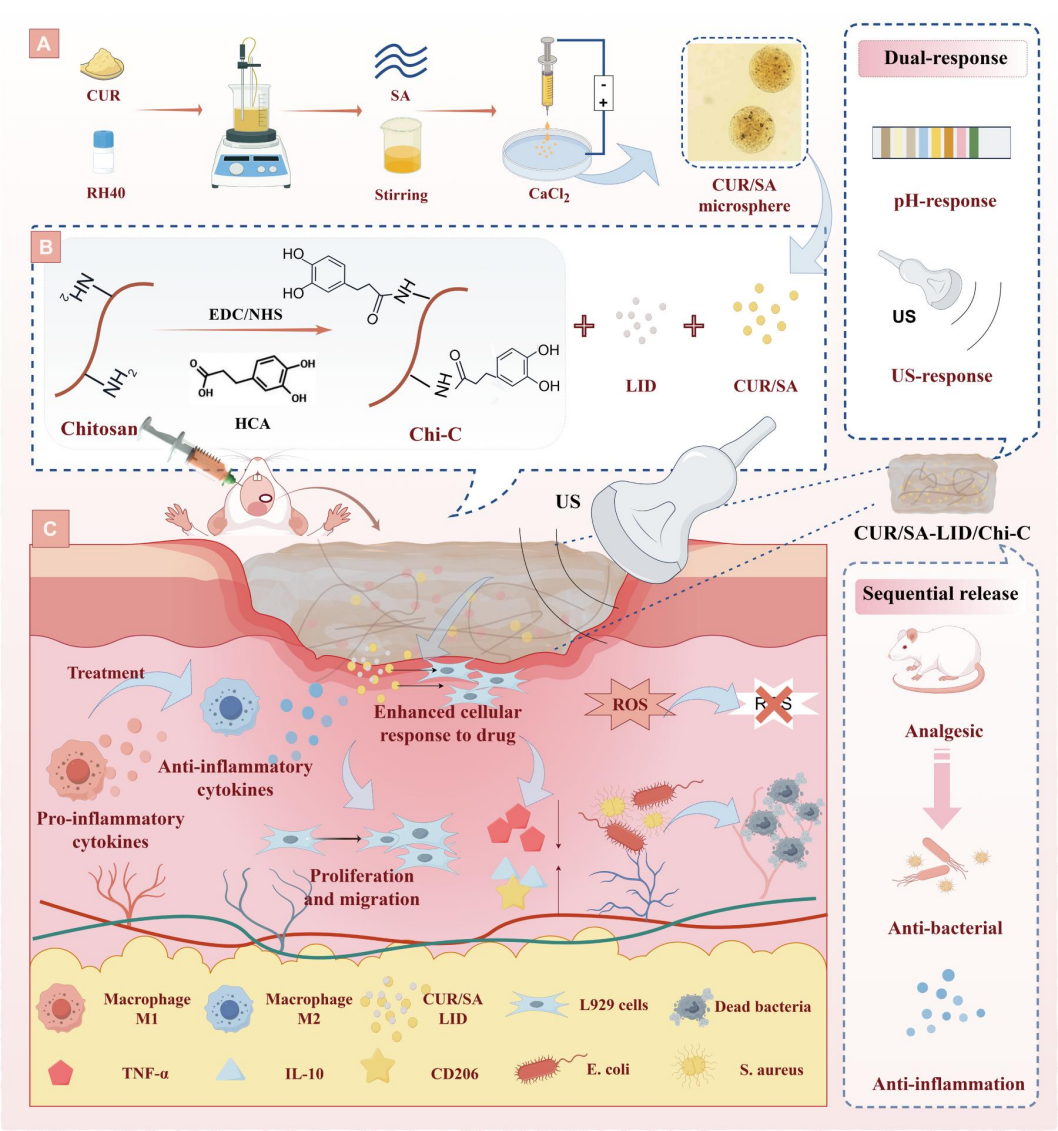
(**A**) Schematic depiction of the synthesis process of CUR-loaded CUR/SA microspheres. (**B**) Schematic illustration of the preparation process of CUR/SA-LID/Chi-C. (**C**) Diagram showing the promotion of infectious oral ulcer healing using CUR/SA-LID/Chi-C+US. Figure 1 was created using Figdraw (www.figdraw.com).

**Figure 2:**
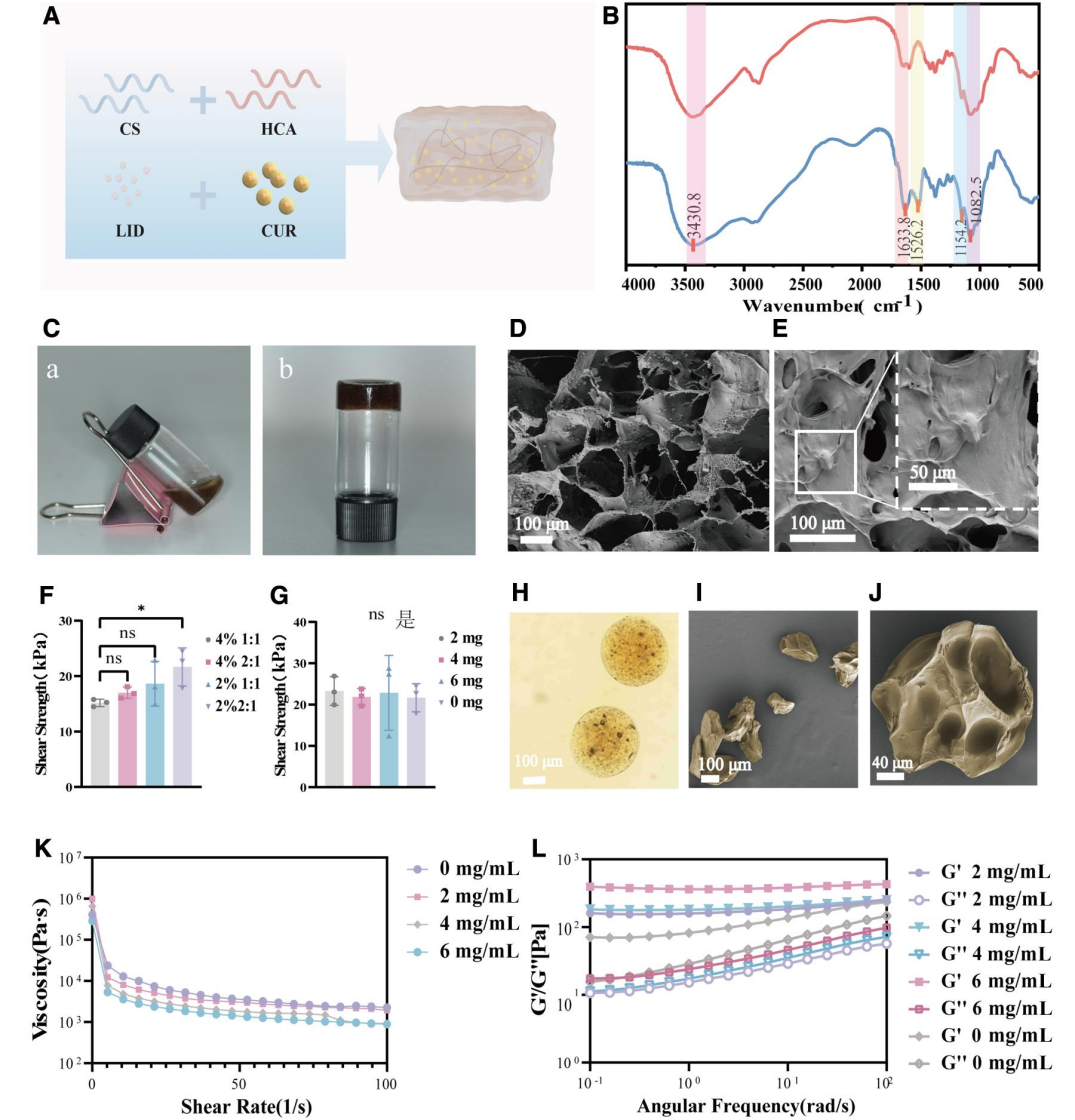
(**A**) Schematic synthesis of CUR/SA-LID/Chi-C (created with Figdraw). (**B**) FTIR spectra of CS and Chi-C. (**C**) Digital photographs of CUR/SA-LID/Chi-C before and after gelation. (**D**) SEM images of Chi-C. (**E**) SEM images of CUR/SA-LID/Chi-C. (**F**) Lap shear strength tests of Chi-C with different proportions. (**G**) Shear rheological tests of CUR/SA-LID/Chi-C after incorporation of different ratios of CUR/SA microspheres. (**H**) Optical microscopy image of CUR/SA microspheres. (**I, J**) Freeze-dried SEM images of CUR/SA microspheres. (**K**) Steady-state shear tests of CUR/SA-LID/Chi-C with different CUR/SA ratios. (**L**) Variation of *G*′ and *G*″ as a function of angular frequency for CUR/SA-LID/Chi-C with increasing CUR/SA content. *P < 0.05 (*n* = 3).

Chi-C hydrogels were synthesized through the previously described method (Figure 1). The two independent variables, polymer concentration (1%, 2%, 4% w/v) and NaIO4/HCA molar ratio (1:1, 1:2), were systematically adjusted. Gelation efficacy exhibited concentration-dependent behavior. No macroscopic gel formation occurred at 1% Chi-C, whereas increased polymer concentration (2–4%) significantly accelerated gelation kinetics (Figure 2C). At constant polymer concentration (2%), elevated NaIO_4_ content reduced gelation time. The research demonstrated that higher polymer concentrations (2–4%) promoted rapid gelation, while increased NaIO_4_ content at constant Chi-C levels further accelerated the gelation process. This underscores the oxidant-dependent efficiency of the crosslinking reaction, which is consistent with observations reported for other catechol-modified polymers [35].

Given the dynamic oral environment, mucoadhesive strength was quantified using pig skin tissue. Grafting of catechol functionalities onto CS enhanced both polymer solubility and interfacial adhesion. The lap shear test revealed a positive correlation between catechol content and adhesive strength, reaching maximum bonding strength (21.15 ± 3.3 kPa) at 2% Chi-C with 1:2 oxidant ratio (Figure 2F). This improvement correlated with increased catechol density and periodate-driven oxidation to quinones, which facilitated covalent bonding with mucosal thiol or amino groups. The mucoadhesive strength of hydrogels is a critical parameter for their application in the oral cavity, where effective and prolonged adhesion is essential for local drug delivery and wound healing. Compared with the results of this study, mucoadhesive strengths reported for other hydrogels in the literature are different from those found in this study.

For example, conventional polyvinyl alcohol (PVA) hydrogels generally exhibit mucoadhesive strengths ranging from 5 to 15 kPa [36], while certain advanced hydrogels, such as those based on polydopamine or with high functional group density, have been reported to attain values over 20 kPa [3, 37]. However, at 4% polymer concentration, bonding strength decreased by approximately 20% despite higher catechol content. This reduction may be explained by the phenomenon of overcrosslinking [38]. Therefore, hydrogel mucoadhesive performance depends not only on surface functional group density but also on the internal network structure and mechanical properties.

### Fourier transform infrared spectroscopy

FTIR spectroscopy confirmed the structural modification of CS through catechol functionalization (Figure 2B). CS exhibits characteristic absorption peaks in the range of 1154–1082 cm^−1^, attributed to the stretching vibrations of C–O bonds in the molecule. The characteristic peak at 1633 cm^−1^ indicates the presence of amide bonds in CS, while the broad peak at 3430 cm^−1^ originates from the hydrogen bond network formed between intermolecular N–H and O–H groups [39]. Comparing the spectra of the Chi-C composite reveals that the spectral lines in the main functional group regions are highly consistent. Chi-C exhibits a characteristic absorption band at 1526 cm^−1^ that is not observed in the CS spectrum. This peak position highly matches the skeletal vibration of the C=C bond in the phenol group, confirming that the phenol group has been successfully grafted onto the CS molecular chain [38]. The introduction of catechol groups brings new structural units to the CS molecules, endowing them with enhanced interfacial reactivity and gelation capability.

### Synthesis and characterization of CUR/SA microspheres

Electrospray technology successfully generated CUR/SA microspheres, achieving an encapsulation efficiency of 82.83 ± 2.34% and a drug loading capacity of 24.64 ± 2.9%. Notably, these values are superior or comparable to many previously reported microsphere systems. For example, studies on CS-based or poly(lactic-co-glycolic acid) (PLGA) microspheres often report encapsulation efficiencies ranging from 60% to 75% and drug loading capacity below 20% [40–42]. Optical microscopy revealed that the as-prepared microspheres exhibited smooth surfaces and uniform sizes (Figure 2H). SEM characterization revealed significant morphological alterations in the microspheres following freeze-drying. Post-lyophilization microspheres exhibited irregular, collapsed structures with pronounced surface wrinkling (Figure 2I and J), contrasting sharply with the spherical morphology observed prior to lyophilization. This structural deformation is attributed to heterogeneous volume contraction during dehydration, resulting in nonuniform stress distribution across the polymeric matrix [43].

### Characterization of CUR/SA-LID/Chi-C hydrogel-microsphere scaffolds

SEM analysis revealed that the Chi-C and CUR/SA-LID/Chi-C scaffolds exhibited a characteristic three-dimensional network structure (Figure 2D). At higher magnifications, microspheres could be observed embedded within the hydrogel matrix (Figure 2E). Rheological characterization of Chi-C hydrogels incorporated with 2 mg LID and CUR/SA microspheres (2–6 mg) demonstrated consistent viscoelastic behavior across all formulations. Storage modulus (*G*′) exceeded loss modulus (*G*″) confirming the formation of a stable, three-dimensional crosslinked network. Importantly, as the microsphere ratio increases, the storage modulus (*G*′) of the scaffold increases (Figure 2I), suggesting that CUR/SA microspheres act as mechanical reinforcements within the scaffold and directly contribute to enhanced structural integrity [44]. All composites retained shear-thinning behavior across shear rates of 0.1–100 s^−1^ (Figure 2H), indicating that their injectability is preserved regardless of microsphere loading. This property is critical for clinical applications, allowing the hydrogel to be easily delivered and molded to irregular sites while maintaining its function [45].

The lap shear test revealed good adhesive strength for CUR/SA-LID/Chi-C composites, showing no statistically significant reduction versus microsphere-free controls (Figure 2G). The mechanism likely involves the synergistic interaction between the three-dimensional hydrogel matrix and the embedded microspheres. The crosslinked network efficiently disperses mechanical stress, while the microspheres provide additional rigidity without disrupting adhesive performance or injectability.

### Responsive release of CUR/SA-LID/Chi-C hydrogel-microsphere scaffolds

**Figure 3:**
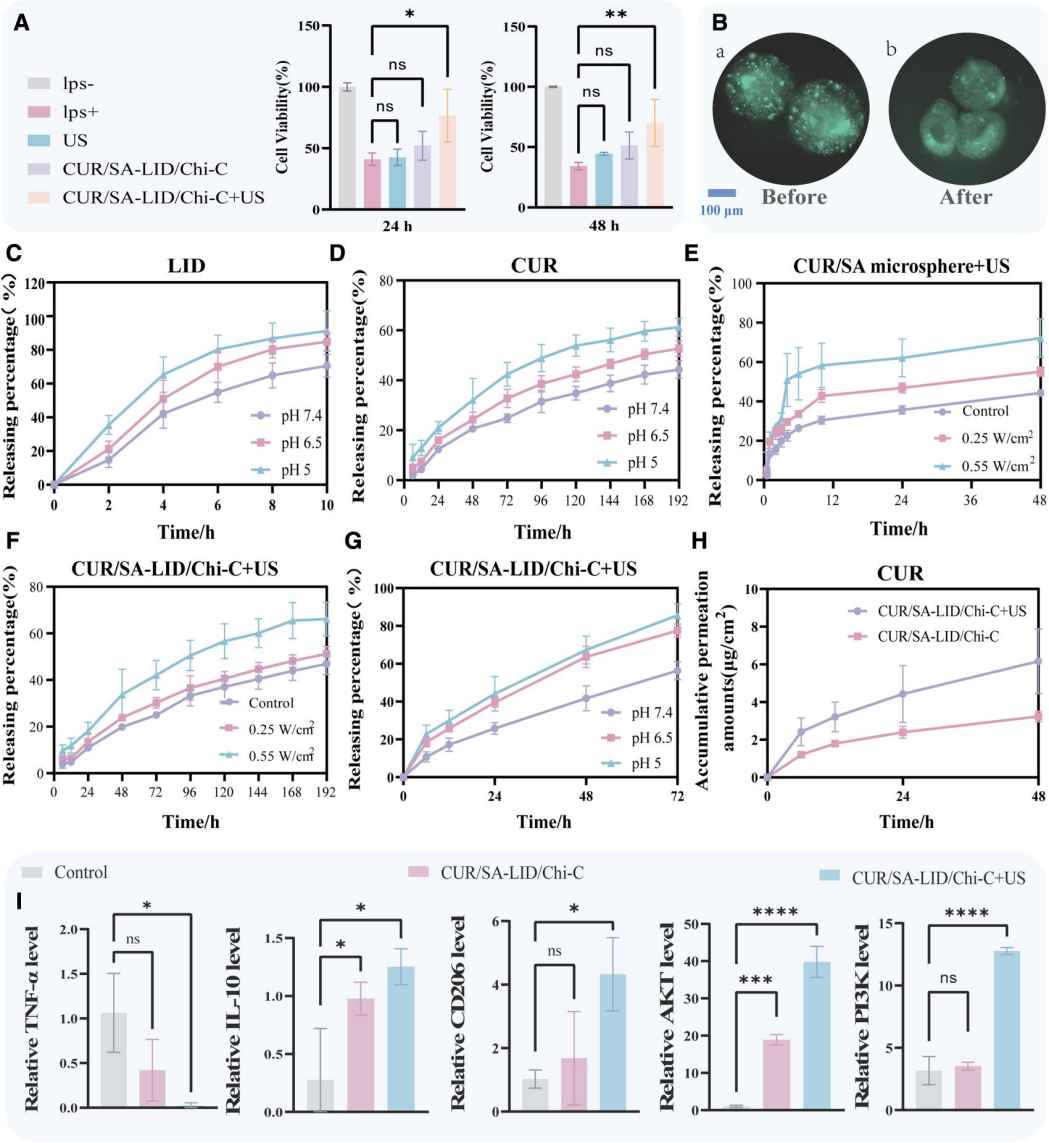
(**A**) RAW 264.7 viability under LPS stimulation after 24/48 h. (**B**) Fluorescence microscopy of CUR/SA microspheres pre- and post-ultrasound. (**C**) LID release from CUR/SA-LID/Chi-C at different pH. (**D**) CUR release from CUR/SA-LID/Chi-C at different pH. (**E**) CUR release from CUR/SA microspheres under varying ultrasound power. (**F**) CUR release from CUR/SA-LID/Chi-C under varying ultrasound power. (**G**) CUR release from CUR/SA-LID/Chi-C+US at different pH. (**H**) Cumulative drug permeation of CUR/SA-LID/Chi-C and CUR/SA-LID/Chi-C+US. (**I**) RT-qPCR results for TNF-α, IL-10, CD206, AKT, PI3K *P < 0.05, **P < 0.01, ***P < 0.001, ****P < 0.0001 (*n* = 3).

The CUR/SA-LID/Chi-C hydrogel-microsphere scaffold demonstrated pH-responsive drug release kinetics, with distinct profiles for LID and CUR. At physiological pH (7.4), LID exhibited rapid release while CUR showed sustained release. Acidic conditions (pH 5.0) significantly accelerated both release profiles. As shown in Figure 3C, acidification from pH 7.4 to 5.0 significantly accelerated LID release within 8 h from approximately 60% to 80%, while doubled CUR release at 12 h. The underlying mechanism for this pH-sensitive behavior is closely related to the swelling properties of the hydrogel matrix. In a weakly acidic environment, the hydrogel swells more extensively, causing the polymer network to expand and facilitating drug diffusion into the surrounding medium [46]. Furthermore, the distinct release profiles of LID and CUR are a designed feature of the scaffold, enabling a programmed, sequential drug release. As quantitatively demonstrated, the initial rapid release of LID is designed for addressing the acute pain at the onset of treatment. This is followed by the deliberately slower, sustained release of CUR, which is crucial for long-term anti-inflammatory and antibacterial effects. This temporal separation, governed by the differential encapsulation of hydrophilic LID within the chitosan network and hydrophobic CUR within the alginate microspheres, directly aligns with the phased therapeutic needs of oral ulcer healing: initial symptom relief followed by prolonged tissue modulation. This pattern, thus, represents a rationally designed therapeutic regimen tailored to the dynamic pathophysiology of the wound.

Additionally, ultrasonic stimulation demonstrated significant control over CUR release kinetics in this composite scaffold. When subjected to acoustic irradiation, selective disruption of ionic crosslinks within the SA matrix occurred via acoustic cavitation, enhancing drug diffusion efficiency [47].

Fluorescence microscopy confirmed ultrasound-induced structural deformation of CUR/SA microspheres, directly correlating with accelerated drug liberation (Figure 3B). For CUR/SA microspheres, baseline cumulative release reached 35.72% at 24 h. Ultrasound intervention (0.55 W/cm^2^, 5 min at *t* = 2 h, 4 h) significantly increased release to 62.09% (Figure 3E). In the CUR/SA-LID/Chi-C composite scaffold, the hydrogel network suppressed initial burst release [48], CUR can only release 10.89% within 24 h. Ultrasound stimulation at 0.55 W/cm^2^ enhanced CUR release to 21.69% (Figure 3F), while 0.25 W/cm^2^ had no significant effect. Thus, ultrasound effectively promotes CUR release from hydrogel-microsphere scaffolds, with the extent controlled by stimulation intensity and duration [49, 50]. This noninvasive approach enhances on-demand and site-specific drug delivery.

Notably, under ultrasound application, the CUR release rate also exhibited significant pH dependence: as the pH decreased from 7.4 to 6.5 and further to 5.0, the release rate accelerated accordingly, reaching the highest level at pH 5.0 (Figure 3G). This finding confirms that our delivery system possesses dual-responsive characteristics to both pH and ultrasound. The acidic microenvironment and the physical effects of ultrasound do not act independently but demonstrate a notable synergistic effect, enabling more efficient and precise ‘on-demand release’ in infectious environments.

### *In vitro* transdermal diffusion study

Franz diffusion cell assays showed that ultrasound significantly enhanced drug transmembrane transport. After 48 h, the hydrogel with ultrasound achieved an approximately 1.94-fold increase in CUR permeation through skin compared to the control without ultrasound (Figure 3H). This confirms that ultrasound not only accelerates drug release from the matrix but also actively promotes its penetration into deeper tissues. The mechanism may involve acoustic cavitation and mechanical stress that transiently disrupt the stratum corneum and induce sonoporation, creating additional pathways for drug molecules [51, 52]. This ultrasound-mediated penetration enhancement, combined with the on-demand release of our scaffold, holds promise for treating deep mucosal lesions.

### *In vitro* anti-inflammatory experiments

Oral ulcer progression involves inflammation, multiple mediators, immune cells and bacterial infection [2, 53]. Modulating biomaterial anti-inflammatory properties aids tissue repair. Following mucosal injury, local inflammation peaks at 24 and 48 h [54]. Given the established efficacy of CUR in alleviating oral mucosal diseases [55], we investigated the regulatory effects of CUR/SA-LID/Chi-C hydrogel microsphere scaffolds on ulcer-associated inflammatory states *in vitro*. Using the LPS-induced RAW264.7 macrophage model, we found that the CUR/SA-LID/Chi-C+US group significantly enhanced cell viability at both 24 and 48 h compared to other groups (Figure 3A).

qPCR was used to assess macrophage-derived cytokine profiles. Relative to the positive control, the CUR/SA-LID/Chi-C+US treatment resulted in decreased TNF-α levels and increased expression of IL-10 and CD206 in macrophages (Figure 3I). These molecular changes are indicative of macrophage polarization from the pro-inflammatory M1 phenotype towards the anti-inflammatory and tissue-reparative M2 phenotype. qPCR also showed upregulated mRNA expression of PI3K and AKT (Figure 3I), providing preliminary transcriptional evidence for the involvement of the PI3K/AKT signaling pathway, which is consistent with the observed M2 polarization phenotype of the cells [56]. It must be pointed out that, although CUR has strong anti-inflammatory activity, its poor pharmacokinetics limit clinical application. Notably, ultrasound not only enhances CUR release and cellular uptake via sonoporation but also exerts its own anti-inflammatory effects, further enhancing the scaffold’s anti-inflammatory response [57]. Emerging evidence indicates that physical therapy with low-frequency ultrasound has been shown to modulate inflammation by activating caveolin-1 and inhibiting MAPK signaling [58], as well as reducing TNF-α and IL-6 expression [59]. The intrinsic bioactivity of CUR, in concert with physical therapy, efficiently suppresses inflammation and facilitates tissue regeneration, thereby improving the therapeutic outcomes in oral mucosal lesions.

### *In vitro* biocompatibility

**Figure 4:**
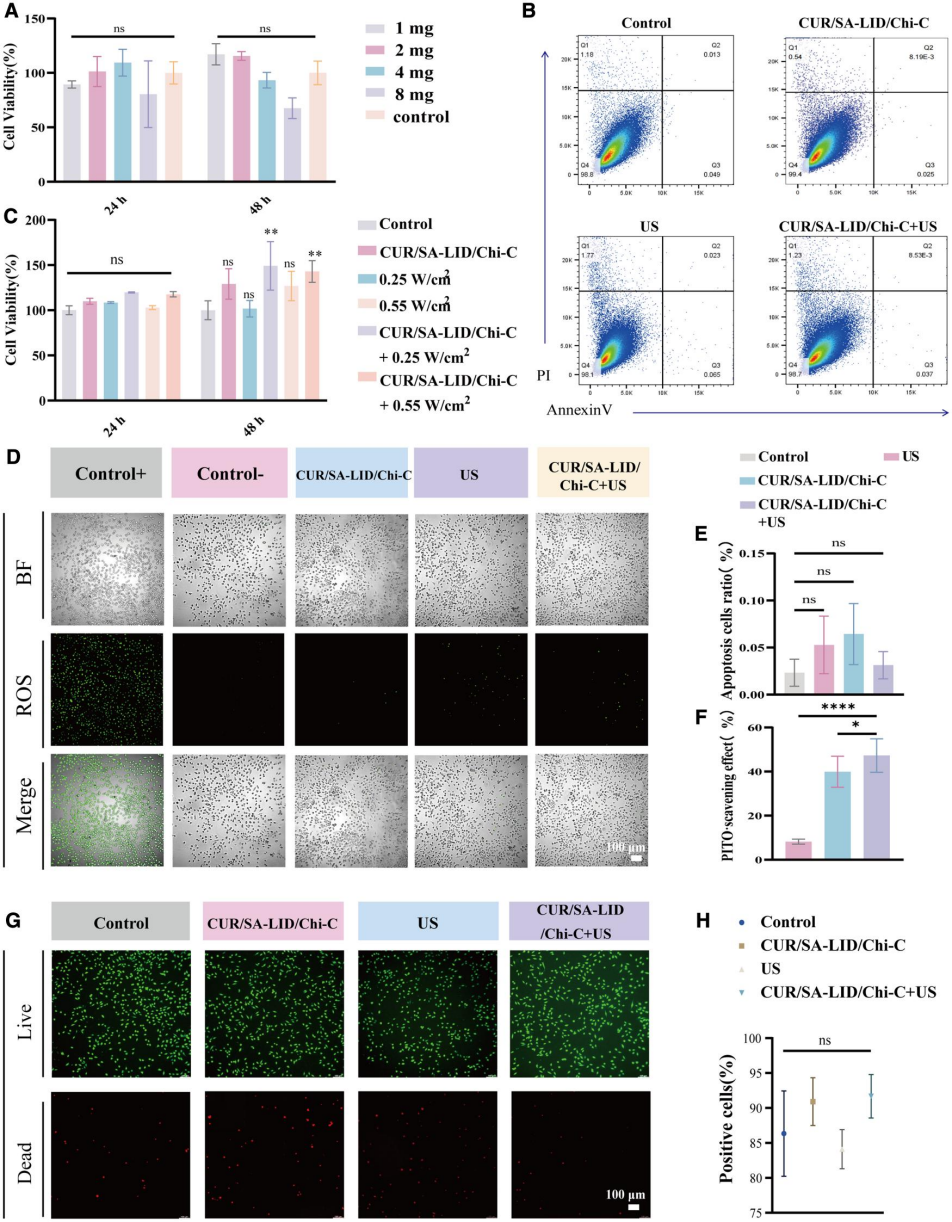
(**A**) Cell viability after 24/48 h with varying CUR/SA microsphere ratios. (**B**) Flow cytometric analysis of L929 cell apoptosis under different treatments. (**C**) Cell viability after 24/48 h with different ultrasound power levels. (**D**) Fluorescence images of intracellular ROS (DCFH-DA staining). (**E**) Quantitative apoptosis analysis. (**F**) PITO· radical scavenging rates. (**G**) Live/dead staining. (**H**) Quantification of live/dead assay. *P < 0.05, **P < 0.01, ****P < 0.0001 (*n* = 3).

The effects of the composite scaffold on cell viability were evaluated using the CCK-8 assay, while LIVE/DEAD staining and flow cytometry-based apoptosis analysis were performed specifically on L929 cells. CCK-8 assay results, obtained using both L929 and HaCaT cells, indicated that at a fixed concentration of LID, a slight reduction in cell viability occurred only when the loading concentration of CUR/SA microspheres reached 8 mg/mL (Figure 4A and Supplementary Figure S1). No significant differences in cell viability were observed in the other experimental groups compared to the control group after 24 and 48 h of incubation.

Notably, L929 cells exposed to ultrasound treatment exhibited enhanced proliferation, which may be attributed to the low-frequency ultrasound mechanical stimulation [60] (Figure 4C). Furthermore, cytotoxicity analyses showed that the CUR/SA-LID/Chi-C group, the US group and the CUR/SA-LID/Chi-C+US group all displayed favorable biocompatibility. LIVE/DEAD staining confirmed most L929 cells remained viable with normal morphology (Figure 4G and H). Annexin V-PI staining showed no significant differences in apoptosis rates compared to the blank control (Figure 4B and E). These findings indicate that the CUR/SA-LID/Chi-C scaffold, with or without ultrasound stimulation, demonstrates good biosafety in L929 cells.

### *In vitro* antioxidant capabilities

Intracellular ROS were monitored using DCFH-DA fluorescence, revealing a significant increase in ROS in the positive control group (1 mM H_2_O_2_), while all treatment groups effectively reduced ROS levels (Figure 4D). Notably, the CUR/SA-Chi-C+US group exhibited the most substantial inhibition, which was corroborated by its highest PITO radical scavenging rate (Figure 4F). The CUR/SA-Chi-C+US group demonstrated significant antioxidant effects. In addition to the direct action of CUR and ultrasound-enhanced drug release [61], we speculate that mechanical stimulation generated by low-intensity ultrasound may also contribute to the regulation of endogenous antioxidant pathways. Previous studies suggest mechanical forces, including ultrasound, can activate the Nrf2-ARE signaling pathway, enhancing antioxidant defense [62]. Moreover, the role of ROS scavenging has recently been highlighted not only in infectious oral mucositis but also in radiation‑induced oral mucositis, where targeted ROS‑clearance strategies show promising therapeutic potential [63, 64].

### *In vitro* cell migration

**Figure 5:**
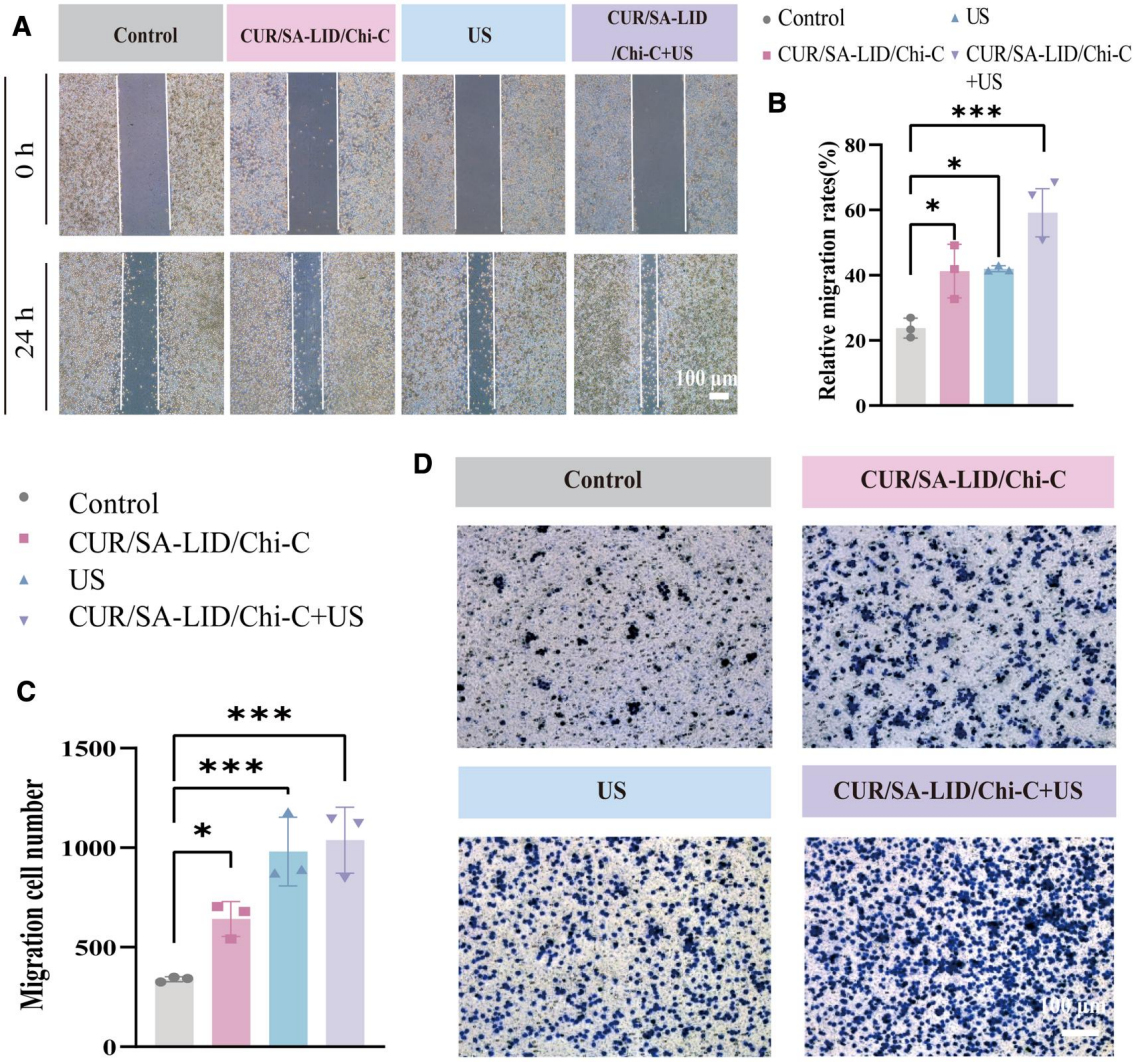
(**A**) Effects of different treatments on L929 cell migration. (**B**) Quantitative analysis of cell migration. (**C**) Quantitative analysis of transwell migration assays. (**D**) Microscopic images of transwell migration assays. *P < 0.05, ***P < 0.001 (*n* = 3).

A chronic inflammatory microenvironment can markedly suppress cellular migration, leading to diminished mucosal cell adhesion and delayed wound healing [65]. This study evaluated the effects of the drug delivery system on L929 fibroblast migration using scratch and Transwell assays (Figure 5). The CUR/SA-LID/Chi-C+US group exhibited significantly higher migration activity than the control group. This improvement likely results from synergistic effects of CUR and ultrasound therapy. Both CUR and low-frequency ultrasound can activate migration-related signaling pathways, including PI3K/AKT and MAPK/ERK [66]. These pathways mediate cytoskeletal rearrangement, cell adhesion turnover and matrix remodeling, orchestrating directed cell movement toward the wound site [19, 66]. Ultrasound may further enhance CUR release, elevating local concentrations to stimulate motility. Additionally, ultrasound mechanical stimulation can directly influence cytoskeletal dynamics and cell movement [67].

### *In vitro* antibacterial experiments

**Figure 6:**
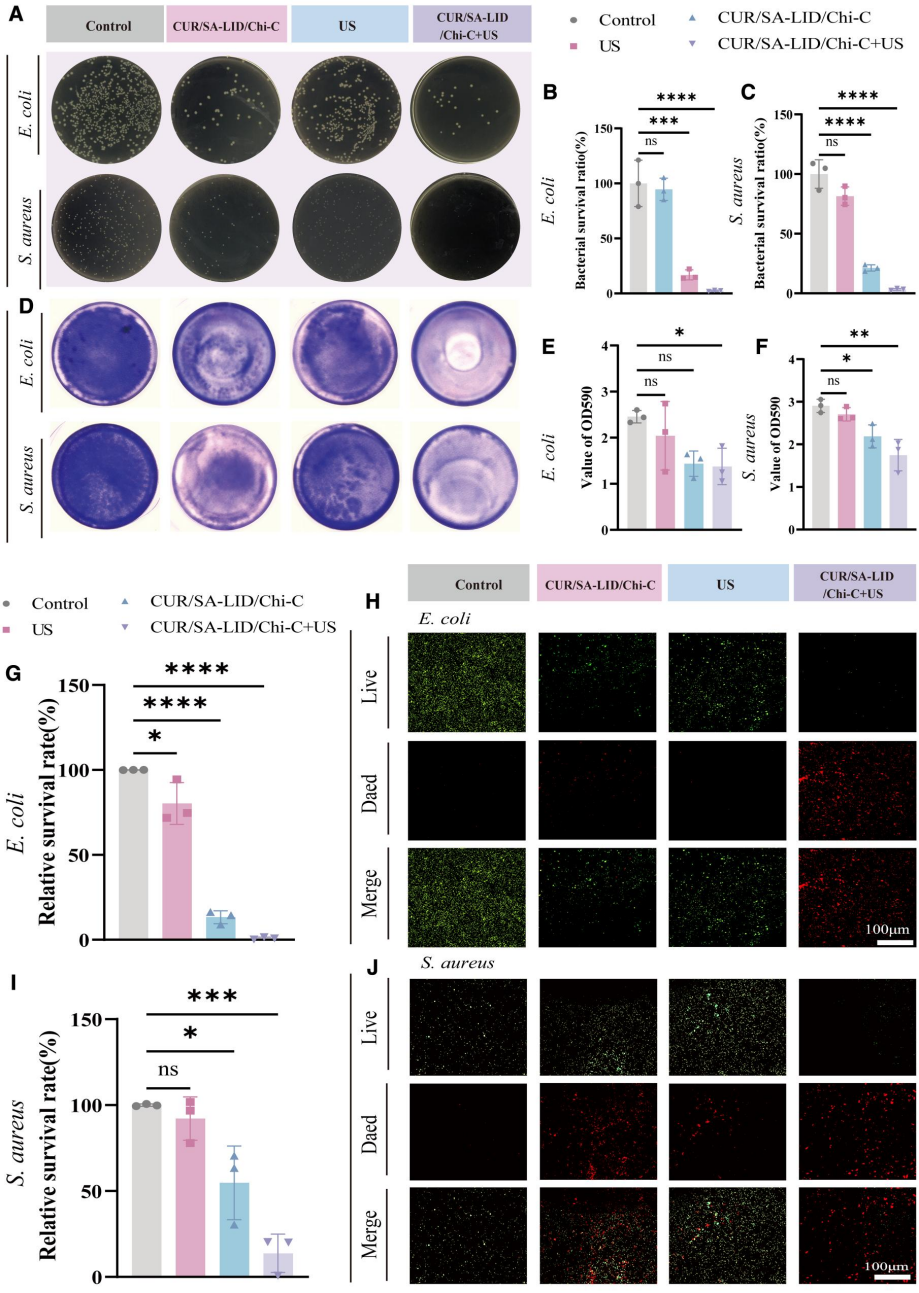
(**A**) Growth images of *E. coli* and *S. aureus* colonies. (**B, C**) Quantitative analysis of *E. coli* and *S. aureus* colonies. (**D**) Crystal violet staining images of *E. coli* and *S. aureus* biofilms. (**E, F**) Quantitative analysis of crystal violet-stained *E. coli* and *S. aureus* biofilms (OD_590_). (**G**) Quantitative analysis of live/dead bacterial staining of *E. coli*. (**H**) Live/dead bacterial staining images of *E. coli*. (**I**) Quantitative analysis of live/dead bacterial staining of *S. aureus*. (**J**) Live/dead bacterial staining images of *S. aureus*. *P < 0.05, **P < 0.01, ***P < 0.001, ****P < 0.0001.

*Staphylococcus aureus* is a common pathogen in oral ulcers, while *Escherichia coli* is often associated with diabetic foot ulcers [68, 69]. Plate counting assays demonstrated that the CUR/SA-LID/Chi-C+US group exhibited the strongest antibacterial effect against both strains under low-intensity ultrasound (Figure 6A–C). Live/dead staining visually confirmed the most potent bactericidal activity in this group (Figure 6H and J); crystal violet staining also revealed its superior biofilm elimination capacity, with biofilm formation lower than other groups. (Figure 6D–F). Notably, ultrasound treatment alone did not exhibit significant bactericidal activity, whereas a pronounced synergistic antibacterial effect was observed when it was combined with the composite scaffold. This synergy is likely attributable to ultrasound-induced sonoporation, a process where acoustic cavitation temporarily increases bacterial cell membrane permeability, thereby improving the uptake and efficacy of the released antibacterial agents from the scaffold [70].

### Mechanisms of CUR/SA-LID/Chi-C in promoting healing under ultrasound stimulation

**Figure 7:**
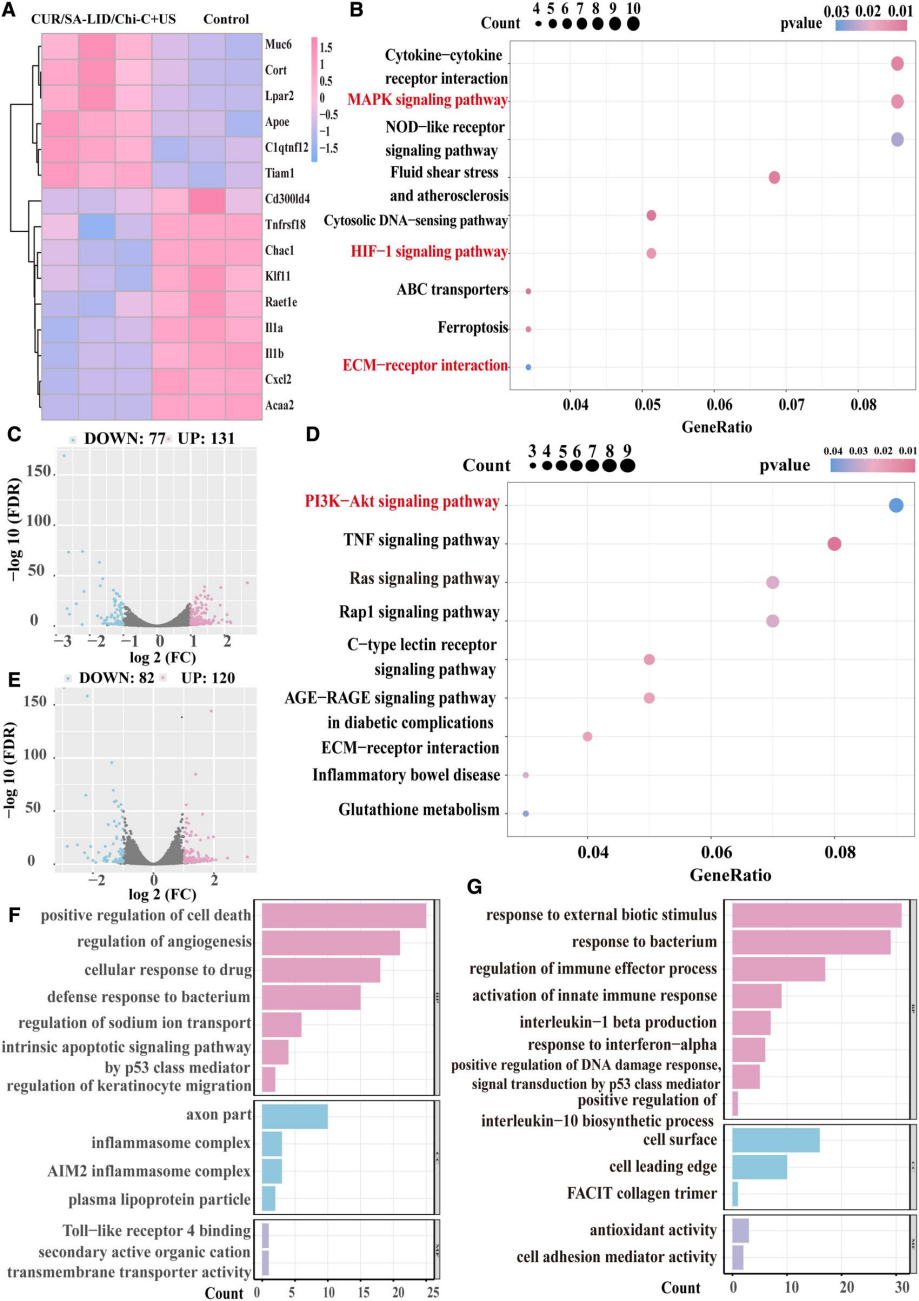
(**A**) Heatmap of selected DEGs (CUR/SA-LID/Chi-C+US vs control). (**B**) KEGG pathway enrichment (CUR/SA-LID/Chi-C+US vs CUR/SA-LID/Chi-C). (**C**) Volcano plot (CUR/SA-LID/Chi-C+US vs CUR/SA-LID/Chi-C). (**D**) KEGG pathway enrichment (CUR/SA-LID/Chi-C+US vs control). (**E**)Volcano plot (CUR/SA-LID/Chi-C+US vs control). (**F**) GO enrichment histogram (CUR/SA-LID/Chi-C+US vs CUR/SA-LID/Chi-C). (**G**) GO enrichment histogram (CUR/SA-LID/Chi-C+US vs control).

To investigate the molecular mechanisms of CUR/SA-LID/Chi-C+US in promoting infectious oral ulcer healing, RNA sequencing was utilized. A total of 202 DEGs were identified between the control and CUR/SA-LID/Chi-C groups and 208 DEGs between the CUR/SA-LID/Chi-C+US and CUR/SA-LID/Chi-C groups (Figure 7A). GO analysis revealed that CUR/SA-LID/Chi-C+US significantly upregulated pathways related to immune activation, innate immunity and responses to bacterial stimuli (Figure 7G). Notably, pathways involved in p53-mediated DNA damage response and synthesis or secretion of inflammatory factors such as IL-1β and IL-10 were markedly affected. p53 pathway activation supports tissue homeostasis and regeneration by preventing accumulation of DNA-damaged cells [71]. KEGG pathway analysis revealed significant enrichment of the PI3K-Akt signaling pathway, a well-established mediator that promotes tissue repair and modulates inflammatory responses [72] (Figure 7D). This finding is consistent with our qPCR results. Mechanistically, PI3K generate phosphatidylinositol-3,4,5-trisphosphate (PIP3), which activates downstream effector AKT, activating AKT to regulate proliferation, migration and metabolism [73]. However, it is important to note that these results are at the transcriptional level; further validation of protein expression and activation is necessary to confirm the pathway’s functional activation. Furthermore, upregulation of antioxidative and reparative genes like Apoe and C1qtnf12 may suggest CUR/SA-LID/Chi-C+US mitigates oxidative stress and enhances repair. The observed downregulation of pro-inflammatory mediators, including Il1b, Cxcl2 and Il1a (Figure 7A), demonstrates effective suppression of excessive inflammatory signaling. Together with the results of qPCR analysis, these findings confirm the anti-inflammatory properties of CUR/SA-LID/Chi-C+US. The resulting attenuation of inflammation establishes a favorable microenvironment for tissue repair, which likely underlies the superior ulcer healing outcomes associated with this therapeutic strategy.

Notably, ultrasound treatment upregulated signaling pathways related to ‘cellular response to drug’ and ‘regulation of keratinocyte migration’ (Figure 7F). These alterations may collectively account for the enhanced drug release, improved anti-inflammatory and antimicrobial efficacy and accelerated ulcer healing observed following ultrasound application. Ultrasound may enhance cellular responsiveness to therapeutics or intrinsic repair signals through mechanisms such as cavitation and mechanical effects [57], offering a promising strategy for enhancing transdermal delivery. For example, sonophoresis can achieve 2- to 6-fold increases in drug penetration [74]. Furthermore, ultrasound may promote tissue repair by establishing a microenvironment conducive to regeneration. Previous studies have shown that low-intensity pulsed ultrasound directly stimulates keratinocyte proliferation and migration, thereby expediting wound closure at the cellular level [75]. Conversely, several inflammation-associated pathways, such as MAPK and HIF-1 signaling, were suppressed (Figure 7B). Additionally, ultrasound stimulation enhanced immune activation features, upregulating the cytosolic DNA-sensing pathway and AIM2 inflammasome to strengthen host defense [76]. Ultrasound-induced microenvironmental changes activate cytoskeleton-related pathways, such as ECM-receptor signaling, which enhance intracellular signal transduction, promote actin cytoskeletal remodeling and increase the activity of adhesion molecules [77]. These findings are consistent with previous observations in cell migration assays. Moreover, the concurrent downregulation of ferroptosis and AGE-RAGE signaling may further support ultrasound’s role in alleviating oxidative stress and hypoxic injury [78]. By reducing iron-dependent cell death and inflammatory responses, ultrasound may create a more stable microenvironment that supports tissue repair.

### Effect of CUR/SA-LID/Chi-C+US on mucosal healing in rats

**Figure 8:**
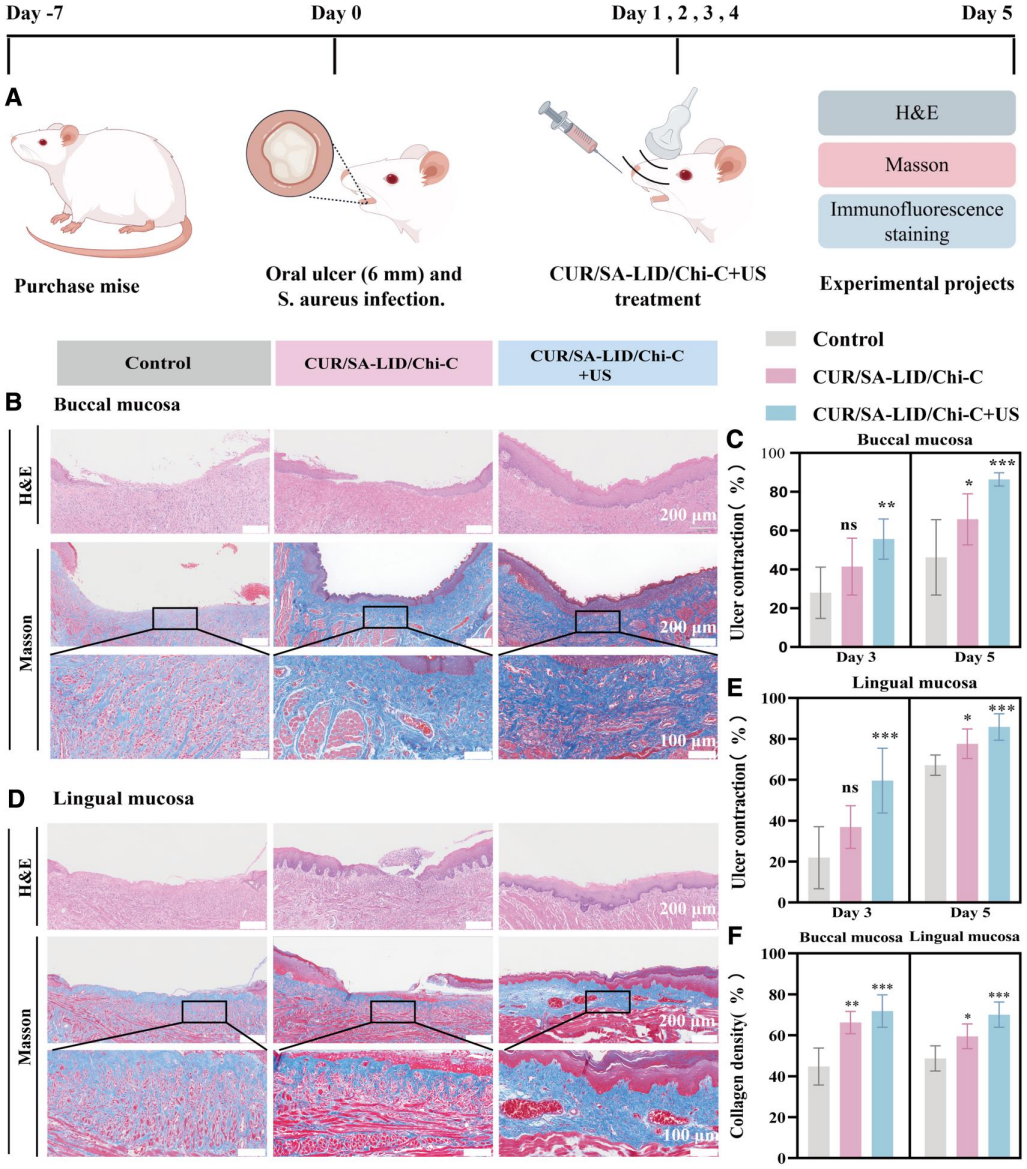
(**A**) A schematic illustration of the protocol for animal experiments. Created with Figdraw (www.figdraw.com). (**B**) H&E and Masson trichrome staining of buccal mucosa ulcers after different treatments. (**C**) Quantitative analysis of daily buccal ulcer healing. (**D**) H&E and Masson trichrome staining of tongue mucosa ulcers. (**E**) Quantitative analysis of daily tongue ulcer healing. (**F**) Quantitative analysis of collagen deposition in ulcer tissues. *P < 0.05, **P < 0.01, ***P < 0.001 (*n* = 5).

**Figure 9:**
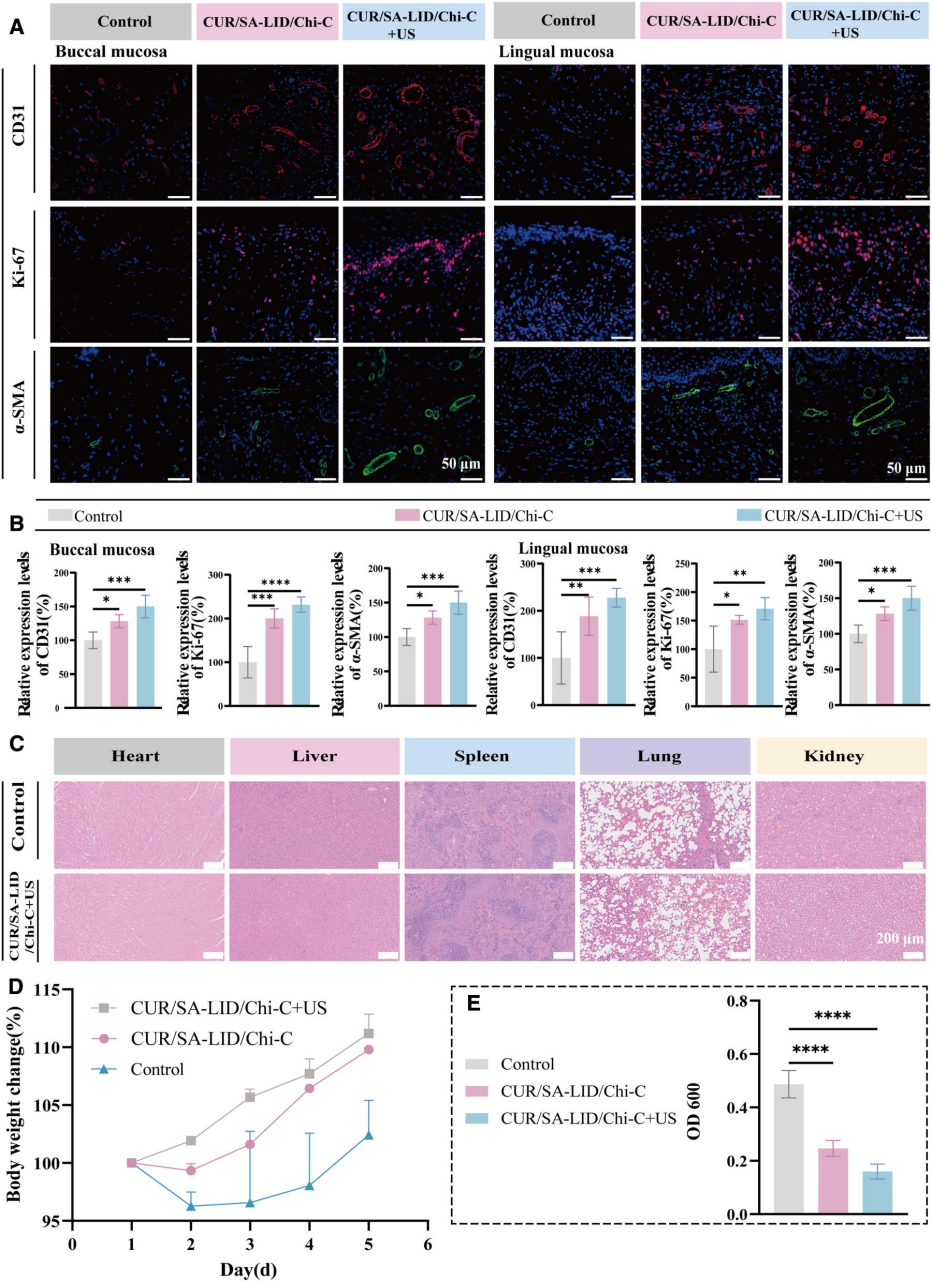
(**A**) Immunofluorescence staining of CD31, Ki-67 and α-SMA in buccal and lingual ulcers. (**B**) Relative expression levels of CD31, Ki-67 and α-SMA. (**C**) H&E staining of major organs (heart, liver, spleen, lung, kidney). (**D**) Body weight change curves of SD rats. (**E**) OD600 values of bacterial samples from infectious ulcers. *P < 0.05, **P < 0.01, ***P < 0.001, ****P < 0.0001 (*n* = 5).

A rat model of oral mucosal defect (6 mm^2^) infected with *Staphylococcus aureus* (1 × 10^8^ CFU·mL^−1^ 10 μL) was established to evaluate reparative efficacy. Longitudinal observations showed that both CUR/SA-LID/Chi-C and CUR/SA-LID/Chi-C+US groups significantly accelerated wound contraction and epithelial regeneration on Day 3 and 5 compared to controls (Supplementary Figure S2). Histopathological analysis confirmed that the scaffold groups promoted granulation tissue formation and angiogenesis, facilitating ulcer healing (Figure 8B and D). H&E staining revealed more continuous and structurally integrated neoepithelium in the treated wounds. Significantly, oral ulcer lesions are often accompanied by the formation of a pseudomembrane, which acts as a physical barrier to drug penetration [79]. While our *in vitro* Franz cell data suggest that ultrasound may help overcome such barriers, these models lack the complexity of the *in vivo* environment. To address this, future work should employ animal models of oral ulcer to directly visualize drug penetration and distribution within deep mucosal tissues under ultrasonic intervention. Furthermore, H&E staining of major organs confirmed no observable toxicity, supporting the excellent biosafety and biocompatibility of our CUR/SA-LID/Chi-C+US system (Figure 9C).

To further clarify the mechanisms underpinning tissue regeneration, we compared collagen synthesis and neovascular density during oral ulcer healing. Masson’s trichrome staining confirmed that both CUR/SA-LID/Chi-C and CUR/SA-LID/Chi-C+US groups promoted collagen fiber deposition, with the latter showing the most pronounced accumulation, indicating strong extracellular matrix remodeling (Figure 8B and D). Additionally, immunofluorescence of CD31 and α-SMA demonstrated enhanced angiogenesis and vessel maturation in both treatment groups, particularly with ultrasound activation. Furthermore, Ki-67 immunofluorescence on Day 5 post-treatment confirmed markedly elevated cellular proliferation in the CUR/SA-LID/Chi-C+US group (Figure 9A and B). The observed enhancement in cell migration can be attributed to the combined anti-inflammatory and pro-regenerative properties of CUR, the efficient release of the drug from the scaffold and the facilitative effects of ultrasound, which together promote greater cellular uptake of the drug and stimulate angiogenic signaling.

Bacteriological analysis of wound samples collected 24 h post-treatment revealed a significant reduction in bacterial load in both the CUR/SA-LID/Chi-C and CUR/SA-LID/Chi-C+US groups compared to the control group, as indicated by decreased OD600 of wound exudates after 24 h incubation in LB medium at 37°C (Figure 9E). While OD600 reflects overall bacterial growth, it does not directly quantify viable bacteria. Future studies will employ CFU counts from homogenized wound tissues to precisely determine bacterial burden *in vivo*, providing a more robust assessment of antibacterial efficacy in the rat oral mucosal infection model.

Longitudinal monitoring of body weight revealed that animals in the control group experienced reduced food intake and consequent weight loss at 48 h post-ulcer induction. In contrast, rats treated with either CUR/SA-LID/Chi-C or CUR/SA-LID/Chi-C+US exhibited stable weight gain after treatment initiation (Figure 9D), likely reflecting reduced lesion-induced discomfort and faster recovery.

Overall, collagen deposition, enhanced angiogenesis, promoted cellular proliferation and infection control, together with improved systemic recovery, indicate the potential advantages of the CUR/SA-LID/Chi-C+US system in infectious oral ulcer healing.

## Conclusion

In this study, we developed a dual-responsive CUR/SA-LID/Chi-C hydrogel–microsphere scaffold for infectious oral ulcer therapy, based on a multistage therapeutic strategy. The platform executes a programmed sequential drug delivery, which was quantitatively validated: an initial rapid release of LID, followed by a sustained release of CUR. Furthermore, ultrasound acts as a potent external trigger. Beyond its established role in accelerating drug release and penetration, ultrasound actively promotes cell proliferation and migration in synergy with therapeutic agents. This pro-regenerative activity, combined with the potential to augment the drug’s antibacterial effects, underscores the significant potential of ultrasound to improve overall therapeutic outcomes. Mechanistic studies further revealed that this strategy modulates both the PI3K-Akt and inflammasome pathways. This work presents a promising approach for personalized infectious oral ulcer treatment, with potential for clinical translation. Future studies will focus on validation in large animal models and further investigation of the underlying molecular mechanisms at the protein level.

## Supplementary Material

rbag063_Supplementary_Data
