# Supplementary material for: Sequential drug release from dual-responsive scaffold with ultrasound-enhanced efficacy for infectious oral ulcer therapy
Source: Regen Biomater. 2026 Mar 26;13:rbag063. doi: 10.1093/rb/rbag063 (PMC13154417; doi:10.1093/rb/rbag063)
Supplement: rbag063_Supplementary_Data [file rbag063_supplementary_data.zip › Supplementary figure 1&2.docx]

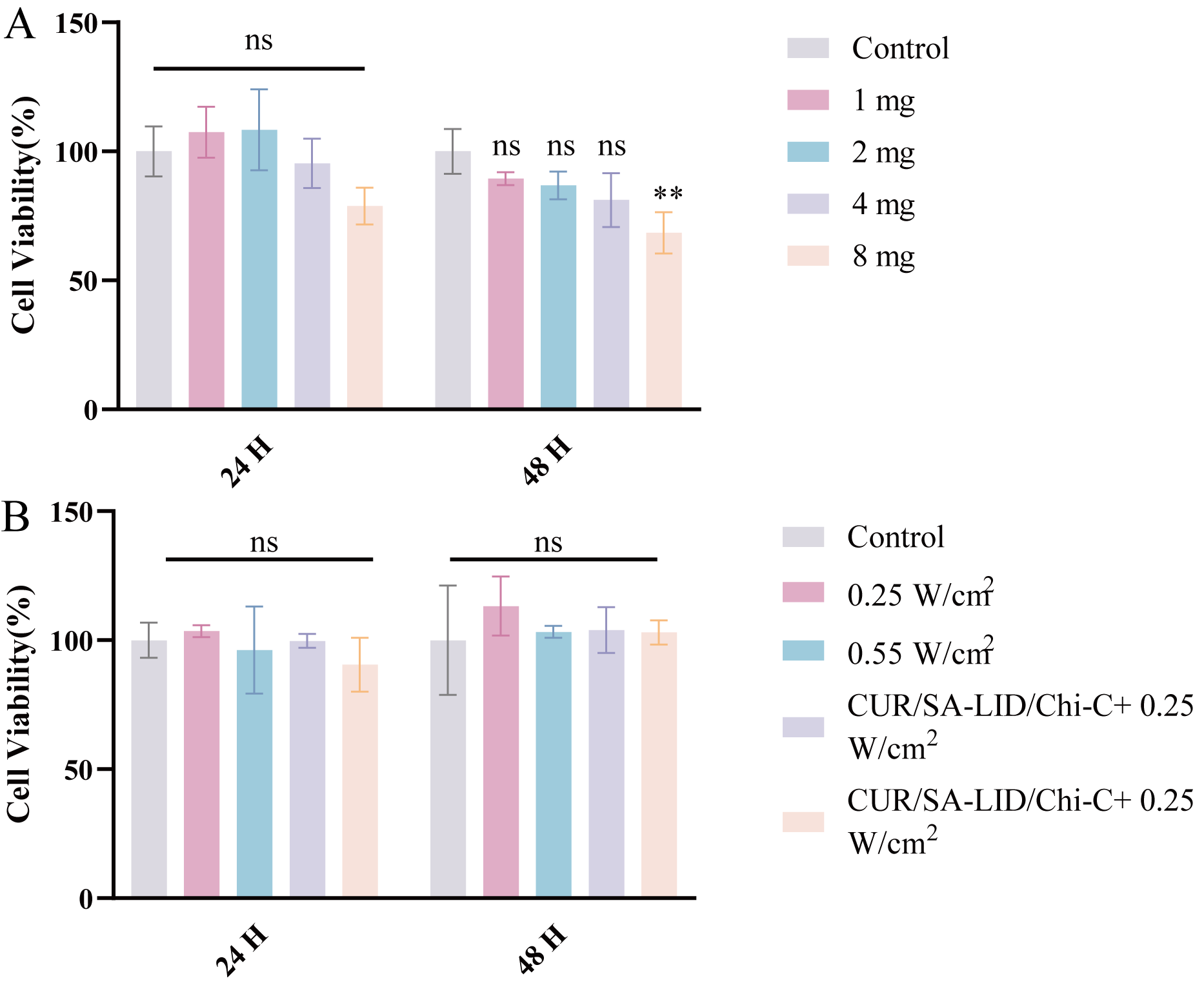


Fig S1 **A.**Viability of HaCaT cells after 24 h and 48 h of treatment with CUR/SA-LID/Chi-C containing varying ratios of CUR/SA microspheres. **B.**Viability of HaCaT cells after 24 h and 48 h of treatment with CUR/SA-LID/Chi-C following the application of ultrasound at different power levels.


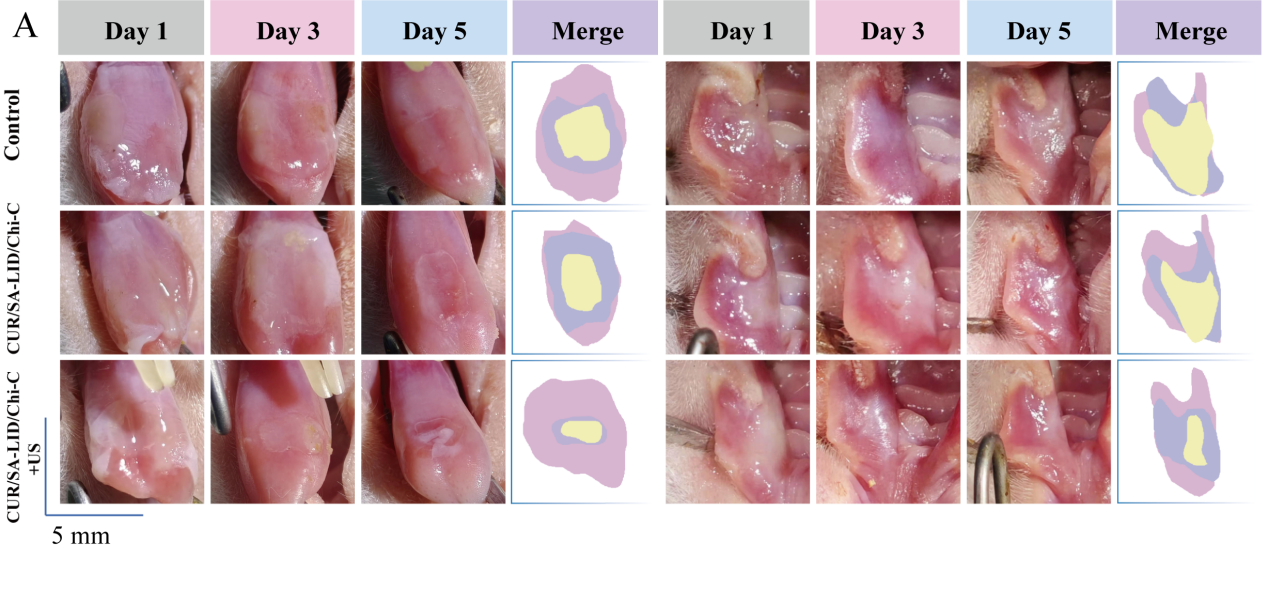


Fig S2 **A.** Photographs of the oral ulcer area at different treatment time points and simulation of the ulcer healing process.
