# Supplementary figures and images for: Sequential drug release from dual-responsive scaffold with ultrasound-enhanced efficacy for infectious oral ulcer therapy
Source: Regen Biomater. 2026 Mar 26;13:rbag063. doi: 10.1093/rb/rbag063 (PMC13154417; doi:10.1093/rb/rbag063)

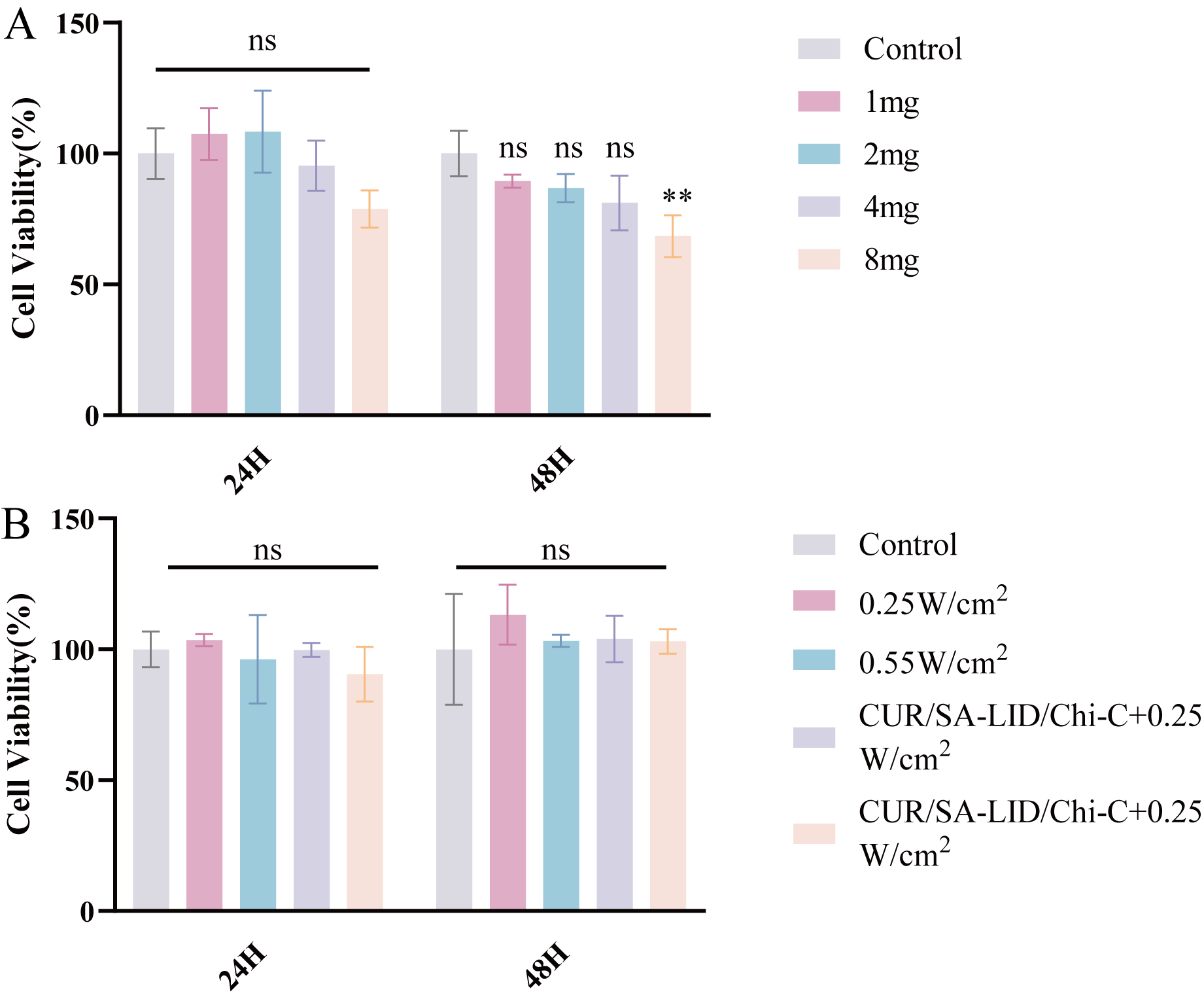

Supplement: rbag063_Supplementary_Data [file rbag063_supplementary_data.zip › Fig S1.tif]

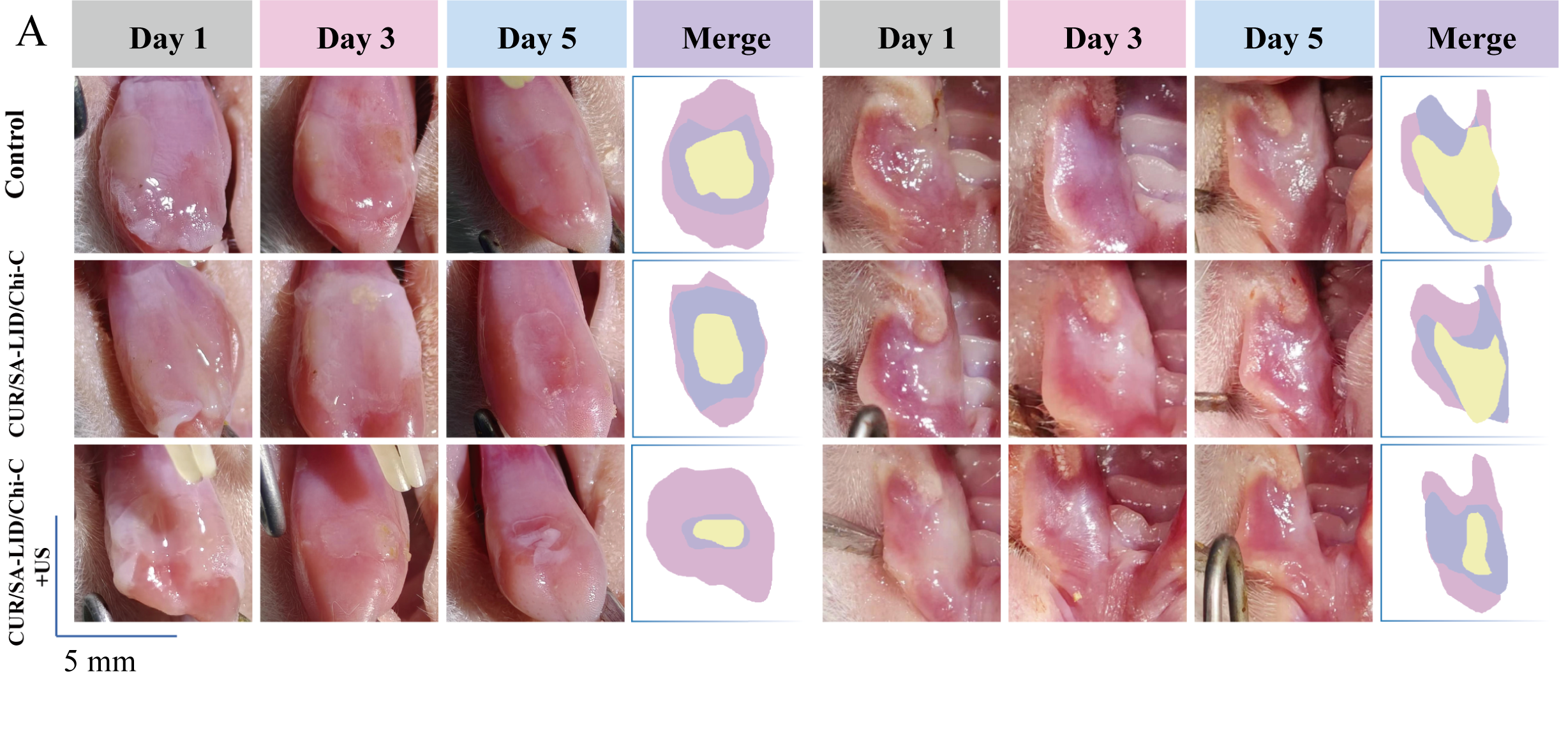

Supplement: rbag063_Supplementary_Data [file rbag063_supplementary_data.zip › Fig S2.tif]
